# Molecular Structures Reveal Synergistic Rescue of Δ508 CFTR by Trikafta Modulators

**DOI:** 10.1126/science.ade2216

**Published:** 2022-10-20

**Authors:** Karol Fiedorczuk, Jue Chen

**Affiliations:** 1 Laboratory of Membrane Biology and Biophysics, The Rockefeller University, New York, NY 10065, USA; 2 Howard Hughes Medical Institute, Chevy Chase, MD 20815, USA

## Abstract

The predominant mutation causing cystic fibrosis, the deletion of phenylalanine 508 (Δ508) in the cystic fibrosis transmembrane conductance regulator (CFTR), leads to severe defects in CFTR biogenesis and function. The advanced therapy Trikafta combines a folding corrector tezacaftor (VX-661), a channel potentiator ivacaftor (VX-770), and a dual-function modulator elexacaftor (VX-445). However, it is unclear how elexacaftor exerts its effects, in part because the structure of Δ508 CFTR is unknown. Here we present cryo-electron microscopy structures of Δ508 CFTR in the absence and presence of CFTR modulators. Elexacaftor partially, when used alone, and fully, when combined with a type I corrector, rectified interdomain assembly defects in Δ508 CFTR. These data illustrate how the different modulators in Trikafta synergistically rescue Δ508 CFTR structure and function.

## One sentence summary

Molecular structures reveal how drugs rectify structure and function of the most common CFTR mutant in cystic fibrosis.

Cystic fibrosis (CF) is a common genetic disease (1) caused by mutations in the gene that encodes the cystic fibrosis transmembrane conductance regulator (CFTR) (2, 3). CFTR is widely expressed in epithelial cells, regulating salt and fluid homeostasis in a variety of tissues. The absence or dysfunction of CFTR causes health issues including malnutrition, liver disease, recurrent bacterial infection, chronic inflammation, and respiratory failure (4).

CFTR belongs to the ATP-binding cassette (ABC) transporter family but functions as an ATP-gated anion channel (5–7). It contains an N-terminal interfacial structure called the lasso motif, two transmembrane domains (TMDs) that form an anion conduction pathway, two cytoplasmic nucleotide-binding domains (NBDs) that bind and hydrolyze ATP (8), and a unique regulatory (R) domain that must be phosphorylated to open the channel (9, 10). Although more than 300 mutations cause CF, approximately 90% of patients carry at least one copy of Δ508 CFTR, in which a single phenylalanine at position 508 is deleted (11, 12). This Δ508 mutant exhibits a severe trafficking defect that results in intracellular retention and premature degradation of the channel (13). Furthermore, the few channels that reach the plasma membrane are unstable and functionally compromised (14–16).

The structure of wild type (WT) CFTR shows that F508 resides on the surface of NBD1, where it makes extensive interactions with the cytosolic region of TM helix 11 and intracellular loop 4 (8, 17). These interactions are critical for both CFTR folding and coupling of ATP-dependent NBD dimerization to pore opening (18), suggesting that disruption of these interactions may underlie both trafficking and functional defects in Δ508 CFTR. Indeed, a previous crystal structure of an isolated NBD1 containing Δ508 revealed a conformation nearly identical to WT NBD1 (19), supporting the hypothesis that Δ508 primarily affects interdomain assembly (8, 19–22). Other studies have shown that lack of F508 causes thermodynamic instability of NBD1, as well as the entire protein (20, 23). Unfortunately, the intrinsic instability of Δ508 CFTR has hindered efforts to structurally characterize the mutant in the context of the entire CFTR protein.

**Fig. 1.**
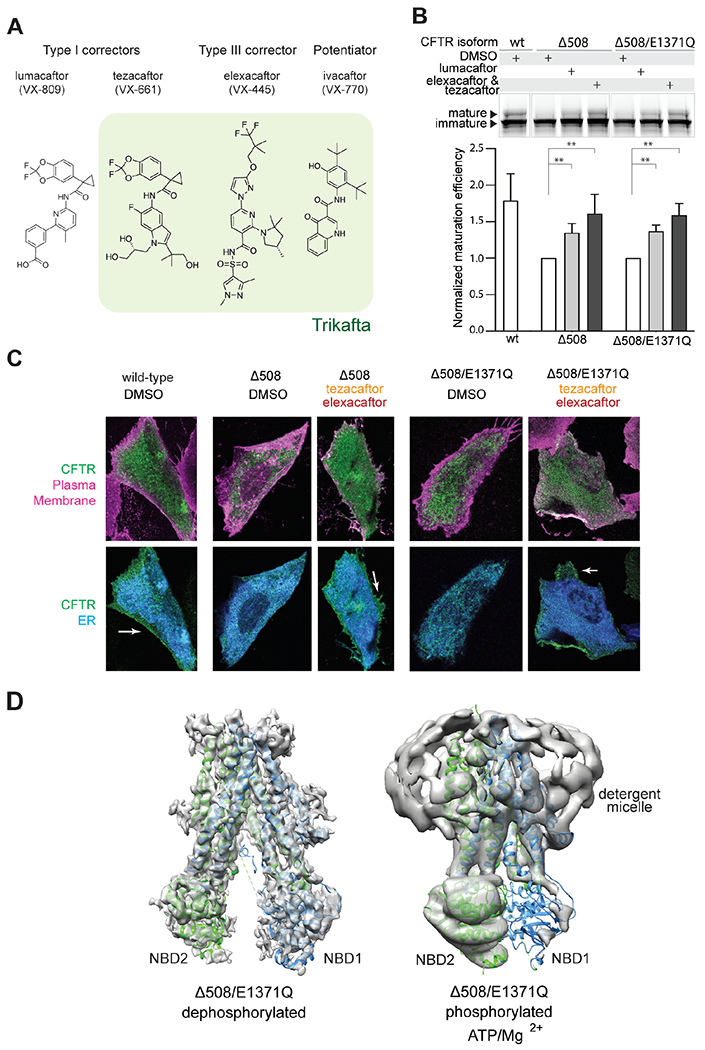
Δ508 CFTR exhibits defective NBD assembly. (**A**) Four CFTR modulators currently used in the clinic. Green highlights the composition of the triple therapy (Trikafta in the US and Kaftrio in the EU). (**B**) Maturation assay in HEK293F cells. Upper panel: SDS-PAGE of cell lysates; mature and immature CFTR were visualized with a C-terminal GFP tag. Lower panel: Quantification of n = 3-6 biological repeats. Standard deviation (SD) indicated by error bars. Lumacaftor, 1 μM; tezacaftor, 10 μM; elexacaftor, 0.5 μM in 0.1% DMSO. Statistical significance calculated using paired t-test. **: 0.001 < p < 0.01. (**C**) Confocal Laser Scanning Microscopy analysis. CHO cells expressing CFTR variants were treated with DMSO (control) or 10 μM tezacaftor and 0.5 μM elexacaftor. ER (blue) visualized by exciting mCherry-tagged Tapasin. Plasma membrane (magenta) visualized by exciting Alexa Fluor 647-conjugated wheat germ agglutinin staining. CFTR (green) visualized by exciting eGFP-tagged CFTR. (**D**) Structures of dephosphorylated and phosphorylated, ATP-bound Δ508/E1371Q CFTR. Cryo-EM maps (grey) are superposed with structures of full-length CFTR in the same conditions (dephosphorylated PDB, 7SVR; phosphorylated, ATP-bound PDB, 7SVD).

Despite these structural obstacles, recently developed CFTR modulators have transformed CF therapy from symptom management to disease correction. These modulators include potentiators that enhance the function of CFTR in the plasma membrane and correctors that increase the presence of CFTR at the cell surface (24, 25). CFTR correctors are further categorized into three different classes based on their functional redundancy (26, 27). Currently, four pharmacological molecules are used in CF therapy, either singly or in combination. These include the potentiator ivacaftor (VX-770), the type I correctors lumacaftor (VX-809) and tezacaftor (VX-661), and the type III corrector elexacaftor (VX-445) (Fig. 1A). The most advanced therapy Trikafta (branded as Kaftrio in the EU) is a combination of ivacaftor, tezacaftor, and elexacaftor. The molecular mechanisms of ivacaftor and type I correctors have been well studied (28, 29). Elexacaftor, however, was discovered only recently and little is known about its mode of action. Interestingly, elexacaftor has been shown to have a dual function, improving CFTR folding as well as ion conductance (27, 30–33), but it is unknown whether it potentiates and corrects via the same binding site.

In this study, we determined cryo-electron microscopy (cyro-EM) structures of Δ508 CFTR and analyzed the molecular effects of lumacaftor (VX-809), tezacaftor (VX-661), and elexacaftor (VX-445), revealing how elexacaftor might potentiate the activity of Δ508 as well as stabilize its structure. We also solved the structure of Δ508 CFTR in the presence of the three modulators comprising the triple therapy, providing a molecular description of how they synergistically rectify Δ508 CFTR to a functional state.

## Δ508 CFTR exhibits defective NBD assembly

We sought to determine the molecular structure of Δ508 CFTR in order to investigate the mechanisms underlying its trafficking defect and gating deficiency. Like the WT CFTR (29, 34, 35), substituting the catalytic residue E1371 with a glutamine was necessary to stabilize an ATP-bound, NBD-dimerized conformation for cryo-EM study. To test whether E1371Q alters the folding of Δ508 CFTR, both with and without pharmacological correctors, we used a gel-shift assay to quantify the relative abundance of the fully glycosylated mature protein relative to the core-glycosylated immature form (36). In the absence of correctors, Δ508 and Δ508/E1371Q CFTR were predominantly in their immature form (core-glycosylated, lower molecular weight). The addition of correctors increased the abundance of mature forms (fully glycosylated, higher molecular weight) for both variants to a similar extent (Fig. 1B). Confocal microscopy confirmed that Δ508 and Δ508/E1371Q CFTR were retained in the endoplasmic reticulum (ER), and that correctors increased their presence at the plasma membrane (Fig. 1C and fig S1). These data demonstrate that similar to Δ508 CFTR, the double mutant Δ508/E1371Q exhibits folding defects that can be reverted by correctors.

Next, we purified the ER-retained Δ508/E1371Q CFTR without any pharmacological correctors (fig. S2) and determined its structure in the absence and presence of phosphorylation and ATP (Fig. 1D). In both conditions, the flexibility of NBD1 became apparent in the initial 2D classification steps of data processing (fig. S3). After extensive 3D classification, the structure of the dephosphorylated, ATP-free form was determined at approximately 6 Å resolution (Fig. 1D and fig. S3), revealing an NBD-separated conformation similar to that of WT CFTR (8, 17). Densities corresponding to the TM helices and NBD2 revealed well-defined secondary structural features. The density for NBD1 was visible but amorphous, with a size and shape consistent with that of NBD1, indicating that Δ508 NBD1 is folded but flexibly attached to the TM helices. This structural observation supports the hypothesis that Δ508 disrupts interdomain assembly (8, 19–22). In contrast to WT CFTR, in which the structured R domain inserts into a cytosolic cleft (8, 17), little density corresponding to the R domain was visible in Δ508 CFTR. As the R domain packs mainly along the surface of NBD1, it is possible that defects in NBD1 assembly also disrupt the correct positioning of the R domain.

The lack of structural stability in Δ508 CFTR was further pronounced in the phosphorylated, ATP-bound conformation. Previous structures of WT, phosphorylated and ATP-bound CFTR carrying the same E1371Q mutation were determined to resolutions between 2.7 and 3.8 Å (29, 34, 35). However, the analogous cryo-EM analysis of Δ508 CFTR stalled at approximately 9 Å resolution due to the heterogenous nature of the particles (Fig. 1D and fig. S2). The overall structure is consistent with an NBD-dimerized conformation, but a notable difference is the absence of visible density corresponding to NBD1 (Fig. 1D and fig. S3). Based on these data, we suggest that the R domain disengages upon phosphorylation as it does in the WT protein, permitting the TMDs to come into close contact. However, in the absence of F508, NBD1 is too flexible to support a stable NBD dimer. Because NBD dimerization is coupled to channel gating in CFTR (37), the inability of the NBDs to dimerize in Δ508 explains its impaired channel function (14–16, 38).

## Correctors restore NBD dimerization in Δ508 CFTR

**Fig. 2.**
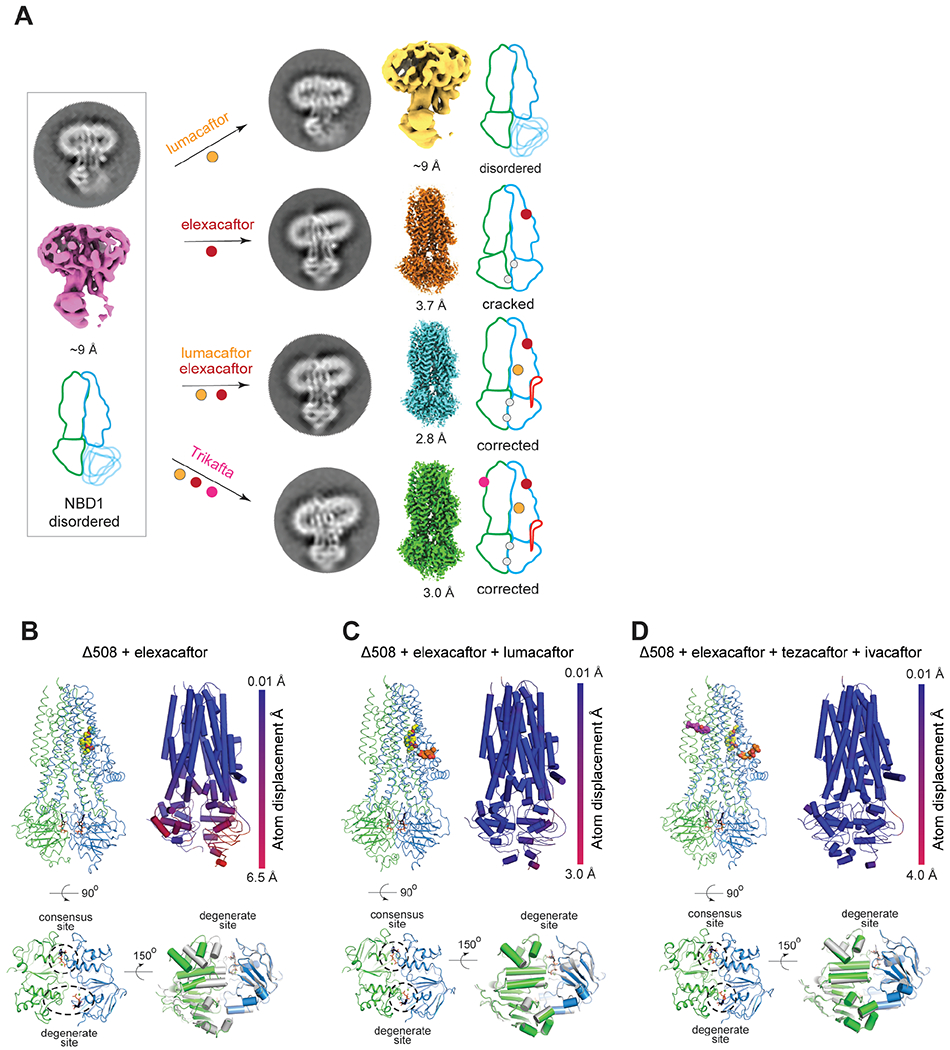
Conformational changes induced by correctors in Δ508 CFTR. (**A**) Summary of structural analysis of Δ508/E1371Q CFTR in the absence and presence of correctors. Average projection from 2D classification, final 3D reconstruction, and schematic representation shown for each structure. NBD1 and TMD1, blue; NBD2 and TMD2, green; R-domain, red; ATP, grey dot; CFTR modulators, colored dots. (**B, C, D**) Structures of Δ508/E1371Q CFTR in complex with modulators. Top left panels: the overall structure. Top right panels: Cα displacements of the Δ508/E1371Q compared to the WT/E1371Q CFTR (PDB, 7SVD). Lower left panels: zoomed-in view of the Δ508 NBD dimer. Lower right panels: superposition of the NBD structures of Δ508 (NBD1, blue; NBD2, green) and WT CFTR (grey). Drug molecules are represented as sphere models.

To investigate the conformational changes that CFTR correctors induce, we performed cryo-EM analyses of phosphorylated, ATP-bound Δ508/E1371Q CFTR in four pharmacological conditions: in the presence of lumacaftor (VX-809); elexacaftor (VX-445); a combination of these two correctors; and the triple Trikafta therapy of ivacaftor (VX-770), tezacaftor (VX-661), and elexacaftor (VX-445) (Fig. 2 and fig. S2 and S3). The Δ508/E1371Q CFTR was expressed in the absence of correctors and solubilized from the ER membrane. Correctors were added during protein purification (fig. S2) to reveal their post-translational effects on the structure of Δ508 CFTR without any confounding effects on folding kinetics and other cellular processes involved in Δ508 biogenesis.

The structure of Δ508 CFTR in the presence of lumacaftor (VX-809) was similar to that in its absence, indicating that post-translational addition of lumacaftor alone is insufficient to correct the structural defects of Δ508 (Fig. 2A). This observation is consistent with the understanding that type I correctors bind to and stabilize TMD1 at an early stage of CFTR biogenesis, preventing its premature degradation and increasing the overall probability of fully folded CFTR being formed (29, 39, 40).

In contrast, the type III corrector elexacaftor (VX-445) stabilized NBD1, resulting in a cryo-EM reconstruction of 3.7 Å resolution with ordered NBDs (Fig. 2A, B). The TMDs of elexacaftor-bound Δ508 CFTR closely resemble those of full-length CFTR in the phosphorylated, ATP-bound conformation (35), but the NBDs are very different (Fig. 2B). Elexacaftor-bound Δ508 has a “cracked-open” NBD dimer in which the catalysis-incompetent (degenerate) site is solvent accessible, and ATP makes contacts exclusively with the NBD1 face of the composite site (Fig. 2B).

The combination of elexacaftor (VX-445) and lumacaftor (VX-809) had an effect that was greater than the sum of each corrector alone, fully restoring Δ508 to an NBD-dimerized conformation with ATP fully bound to both the consensus and degenerate sites (Fig. 2A, C). Moreover, this structure is essentially identical to that obtained in the presence of Trikafta (Fig. 2A), indicating that ivacaftor (VX-770) does not induce further conformational changes. This is consistent with ivacaftor being a potentiator, not a corrector. Both structures closely resemble that of full-length CFTR, having an overall root-mean-square deviation (r.m.s.d) to the full-length protein of 0.5 Å over 1090 Cα positions (Fig. 2C, 2D). We therefore designated both elexacaftor/lumacaftor and Trikafta-bound Δ508 CFTR structures as having a “corrected” conformation, and selected Trikafta-bound Δ508 for further analysis.

## Pharmacologically corrected Δ508 CFTR has a modified NBD1/TMD interface

**Fig. 3.**
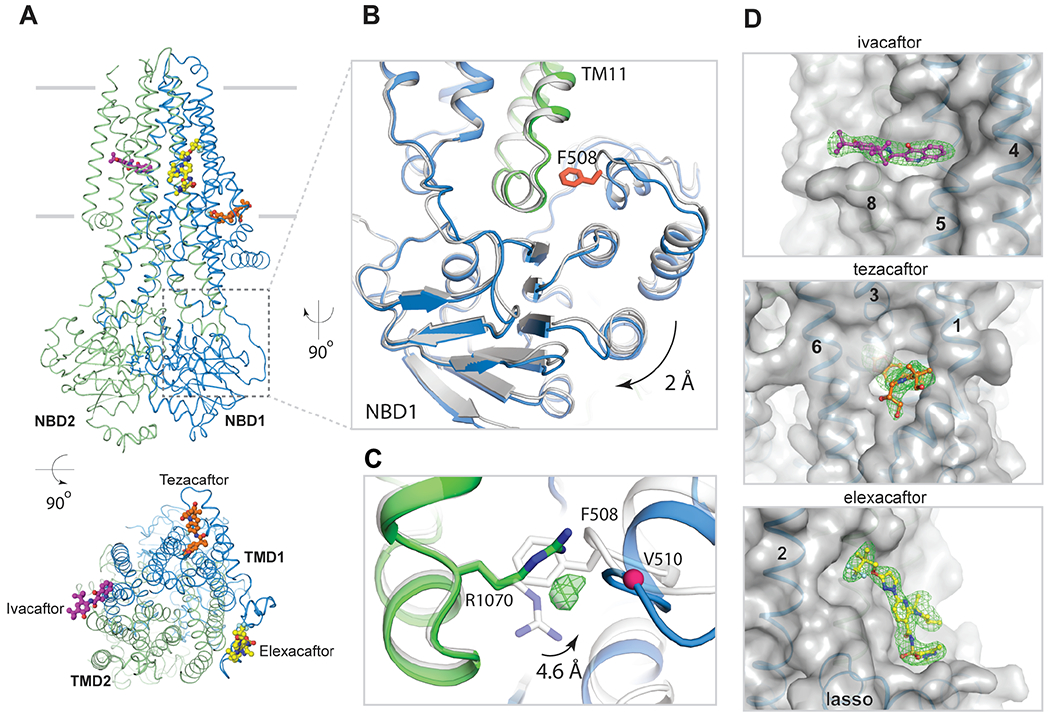
The structure of Trikafta-corrected Δ508 CFTR. (**A**) Orthogonal views of Δ508/E1371Q CFTR in complex with ivacaftor, elexacaftor, and tezacaftor. Δ508 CFTR ribbon diagram with ball-and-stick models of drug molecules. Membrane location indicated by two grey lines. (**B**) Zoomed-in view of F508 site. Δ508 CFTR structure (blue/green) superimposed with full-length CFTR structure (grey). F508 highlighted in red. (**C**) R1070 conformational change. Unassigned density shown as green mesh. (**D**) Zoomed-in views of drug binding sites. Drug densities, green mesh; CFTR, transparent surface model; TM helices and lasso motif as indicated.

The corrected Δ508 structure in the presence of Trikafta differs from that of full-length CFTR in the region of the F508 deletion site (Fig. 3A–C). F508 is located in a loop on the surface of NBD1, projecting its aromatic side chain into a hydrophobic pocket at the end of TM helix 11 (Fig. 3B). Deletion of F508 shortens this loop, leaving a crevice at the NBD1/TMD interface. In addition, the helical subdomain of Δ508 NBD1 (residues 500-564) is shifted away from the interface by approximately 2 Å (Fig. 3B). The crevice at the NBD1/TMD interface is partially filled by R1070, whose side chain swings into contact with main chain atoms in NBD1 (Fig. 3C). We also observed a strong spherical density in the Δ508 cavity area, which may be an ion or water molecule.

Previous work has shown that the V510D mutation can stabilize Δ508 CFTR, likely due to aspartic acid forming a salt bridge with R1070 (41). The structure of Δ508 is compatible with a salt bridge between these residues and thus lends support to this hypothesis (fig. S4A). The structural differences at the NBD1/TMD interface also explain the opposing effects of the R1070W mutation in full-length versus Δ508 CFTR. In full-length CFTR, substituting R1070 with tryptophan inhibits protein folding and leads to CF (20, 42, 43). This is because the large tryptophan side chain at position 1070 would sterically clash with F508 (fig. S4B). In contrast, the same substitution in the Δ508 background partially restores CFTR folding (20, 22, 41, 43, 44), likely due to R1070W strengthening the NBD1/TMD interface by filling the space devoid of F508 and forming hydrophobic and hydrophilic contacts with Δ508 NBD1 (fig. S4C).

## Trikafta modulators bind to distinct sites on Δ508 CFTR

In the cryo-EM reconstruction of Trikafta-bound Δ508 CFTR, the densities for ivacaftor (VX-770), tezacaftor (VX-661), and elexacaftor (VX-445) were strong and unambiguous (Fig. 3). Viewed from the extracellular space perpendicular to the membrane, these compounds form a triangular belt encircling the TMDs (Fig. 3A). The potentiator ivacaftor (Fig. 3, purple molecule) binds to a cleft formed by TM helices 4, 5, and 8, approximately halfway through the lipid bilayer, coincident with the TM8 hinge region involved in gating (28). The molecular details of ivacaftor binding are identical to those in full-length CFTR (28), indicating that ivacaftor potentiates both full-length and Δ508 CFTR via the same mechanism. Similarly, tezacaftor (Fig. 3, orange molecule) binds to Δ508 by inserting into a hydrophobic pocket in TMD1 (Fig. 3D) in a manner identical to that in the WT protein (29). As previously discussed, such a penetrating cavity would cause substantial destabilization of the protein in the absence of tezacaftor (29). Furthermore, TM helices 1, 2, 3, and 6 that form the binding site are predicted to be unstable in the membrane, thus type I correctors would stabilize TMD1 both by filling the cavity and structurally linking together the four unstable helices (29).

**Fig. 4.**
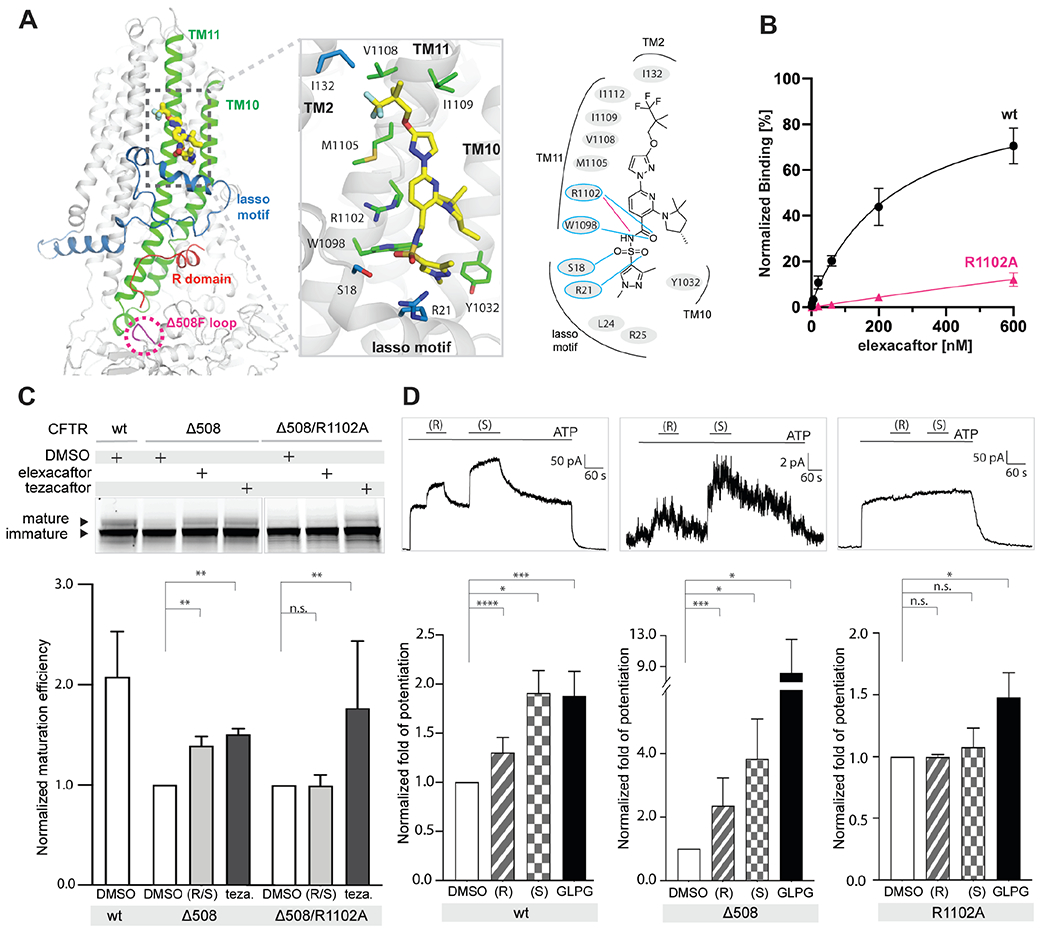
The elexacaftor binding site (**A**) Molecular structure of elexacaftor binding site. Left panel: ribbon diagram. Middle panel: zoomed-in view of molecular interactions. Residues within 4.5 Å of elexacaftor shown as stick models. Right panel: schematic of molecular interactions. Salt bridge, magenta line; hydrogen bonds, blue lines. (**B**) Quantitative measurement of elexacaftor binding. Data points represent mean and SD for n = 6-12 technical (3-4 biological) repeats. Apparent K_d_ of elexacaftor for WT CFTR, 244 ± 50 nM. (**C**) R1102A mutation diminishes correction by elexacaftor. Upper: SDS-PAGE of cell lysates. Lower: Quantification of n = 3 biological repeats. Labels: (R/S), racemic mixture of elexacaftor (0.5 μM); Teza, tezacaftor (10 μM). (**D**) Elexacaftor potentiation. Upper: Representative macroscopic current traces of fully phosphorylated WT and mutant CFTR in inside-out CHO cell membrane patches. Lower: Quantification of n = 3-8 biological repeats. Concentrations used: ATP, 3 mM; elexacaftor, 1 μM; GLPG1837, 10 μM. Fold potentiation normalized to currents with 3 mM ATP in the absence of potentiators. Statistical significance calculated using paired t-test. Labels: not significant, n.s.; p < 0.05, *; p < 0.01, **; p < 0.001, ***; p < 0.0001, ****.

In contrast, the binding site for elexacaftor (VX-445) has not previously been observed. Densities corresponding to elexacaftor are very similar and well-defined in the three elexacaftor-bound reconstructions (elexacaftor alone, elexacaftor with lumacaftor, and Trikafta), enabling an accurate description of elexacaftor’s binding pose and the chemistry of drug recognition (fig. S5A). Elexacaftor (Fig. 3, yellow molecule) binds to CFTR within the membrane, extending from the center of the lipid bilayer to the edge of the inner leaflet (Fig. 4A). The binding pocket is much shallower than that of type I correctors, as if the drug molecule is patched onto the surface of CFTR. Elexacaftor interacts most extensively with TM helix 11 through electrostatic and van der Waals interactions. It also forms contacts with TM helices 2, 10, and the lasso motif (Fig. 4A, fig. S5B).

Studies of ivacaftor (VX-770) binding to CFTR have shown that hydrogen bonds (H-bonds) are critical for drug recognition as they stabilize polar groups in the low dielectric environment of the membrane (28). To test if this principle applies to elexacaftor (VX-445) binding, we substituted R1102 with an alanine to eliminate the formation of an H-bond and a salt bridge (Fig. 4A), and directly measured binding using the scintillation proximity assay (SPA) (Fig. 4B). Specific binding of elexacaftor to full-length CFTR increased as a function of drug concentration. In contrast, the interaction of elexacaftor with the R1102A mutant was barely detectable. We also evaluated the contribution of R1102 to the restoration of Δ508 folding by elexacaftor using the gel-shift assay (Fig. 4C). The R1102A mutation abolished correction by elexacaftor, but not tezacaftor, which binds to a pocket distant from R1102. Finally, to test if R1102 also contributes to the potentiation activity of elexacaftor, we measured macroscopic current in inside-out membrane patches containing phosphorylated CFTR (Fig. 4D). We consistently observed stronger potentiation by the S enantiomer in both WT and Δ508 CFTR, in agreement with the higher efficacy of the S enantiomer observed in prior work (45). However, R1102A CFTR did not respond to either R- or S- enantiomer, indicating that both enantiomers bind to the site identified in the structures. A control potentiator, GLPG1837, increased macroscopic current in all CFTR variants (Fig. 4D). Together, these data suggest that elexacaftor achieves both correction and potentiation via the same binding site.

## Discussion

The structures of Δ508 CFTR reveal that the absence of F508 disrupts the ability of NBD1 to assemble with the TM helices, which leads to intracellular retention and degradation of the protein (14, 20, 46). Furthermore, the inability to form a stable NBD dimer following phosphorylation and ATP binding results in a dysfunctional channel, even if Δ508 CFTR reaches the plasma membrane (14). Pharmacological correctors used in clinic – even added during protein purification– can partially or fully restore the NBD-dimerized conformation. These correctors can improve the folding of WT CFTR as well as disease-causing mutations (47–51). Although it is theoretically possible that correctors salvage the different mutant and WT forms of CFTR by entirely different mechanisms, it is likely that their mechanism of action is the same in all cases. Indeed, lumacaftor (VX-809) binds to Δ508 CFTR and WT CFTR at the same site and by interacting with the same residues. Likewise, ivacaftor (VX-770) interacts with WT and Δ508 CFTR in an identical manner, indicating that its mechanism of potentiation is the same for both WT and mutant CFTR.

The dual-function modulator elexacaftor (VX-445) engages TM helices 10-11 and the lasso motif, all of which are important for CFTR folding and function. Mutations in the lasso motif can cause intracellular retention or abnormal gating, and some lead to CF (52–59). TM10-11 are the “domain swapped” helices of TMD2 that extend into the cytosol and interact with NBD1. This interface is not only important for protein assembly, but also critical for transmitting conformational changes of the NBDs to the TMDs to control ion permeation. Although the details of how elexacaftor potentiates CFTR await incisive electrophysiology measurements, our structural observations indicate that elexacaftor stabilizes TM 10-11, thereby strengthens the TMD/NBD1 interface, which is particularly vulnerable to disease-causing mutations (60).

Recently Braakman and colleagues demonstrated that CFTR folding occurs in a stepwise manner (60). It is likely that the type I corrector tezacaftor binds at an early stage of CFTR biogenesis to stabilize the N-terminal TMD1 (29) and the type III corrector elexacaftor binds at a later stage when TMDs assemble to form a protease-resistant form. Together, these two correctors prevent premature degradation in the ER. Once CFTR reaches the plasma membrane, the presence of elexacaftor strengthens allosteric communication between ATP-bound NBD dimers and the channel gate, thereby increasing ion conductance. Channel activity is further enhanced by ivacaftor, which stabilize the open configuration of the pore (28). In this manner, the three modulators in the triple therapy act synergistically to improve the folding and function of CFTR.

## Supplementary Material

1

## References

[1] Shteinberg M, Haq IJ, Polineni D, Davies JC, Cystic fibrosis. Lancet 397, 2195–2211 (2021). 34090606 10.1016/S0140-6736(20)32542-3

[2] Kerem B , Identification of the cystic fibrosis gene: genetic analysis. Science 245, 1073–1080 (1989). 2570460 10.1126/science.2570460

[3] Riordan JR , Identification of the cystic fibrosis gene: cloning and characterization of complementary DNA. Science 245, 1066–1073 (1989). 2475911 10.1126/science.2475911

[4] Sanders DB, Fink AK, Background and Epidemiology. Pediatr Clin North Am 63, 567–584 (2016). 27469176 10.1016/j.pcl.2016.04.001 PMC4967225

[5] Anderson MP , Nucleoside triphosphates are required to open the CFTR chloride channel. Cell 67, 775–784 (1991). 1718606 10.1016/0092-8674(91)90072-7

[6] Anderson MP, Rich DP, Gregory RJ, Smith AE, Welsh MJ, Generation of cAMP-activated chloride currents by expression of CFTR. Science 251, 679–682 (1991). 1704151 10.1126/science.1704151

[7] Drumm ML , Correction of the cystic fibrosis defect in vitro by retrovirus-mediated gene transfer. Cell 62, 1227–1233 (1990). 1698126 10.1016/0092-8674(90)90398-x

[8] Zhang Z, Chen J, Atomic Structure of the Cystic Fibrosis Transmembrane Conductance Regulator. Cell 167, 1586–1597 e1589 (2016). 27912062 10.1016/j.cell.2016.11.014

[9] Seibert FS , Influence of phosphorylation by protein kinase A on CFTR at the cell surface and endoplasmic reticulum. Biochim Biophys Acta 1461, 275–283 (1999). 10581361 10.1016/s0005-2736(99)00163-7

[10] Ostedgaard LS, Baldursson O, Welsh MJ, Regulation of the cystic fibrosis transmembrane conductance regulator Cl-channel by its R domain. J Biol Chem 276, 7689–7692 (2001). 11244086 10.1074/jbc.R100001200

[11] Amaral MD, Farinha CM, Rescuing mutant CFTR: a multi-task approach to a better outcome in treating cystic fibrosis. Curr Pharm Des 19, 3497–3508 (2013). 23331027 10.2174/13816128113199990318

[12] Riordan JR, CFTR function and prospects for therapy. Annu Rev Biochem 77, 701–726 (2008). 18304008 10.1146/annurev.biochem.75.103004.142532

[13] Cheng SH , Defective intracellular transport and processing of CFTR is the molecular basis of most cystic fibrosis. Cell 63, 827–834 (1990). 1699669 10.1016/0092-8674(90)90148-8

[14] Dalemans W , Altered chloride ion channel kinetics associated with the delta F508 cystic fibrosis mutation. Nature 354, 526–528 (1991). 1722027 10.1038/354526a0

[15] Lukacs GL , The delta F508 mutation decreases the stability of cystic fibrosis transmembrane conductance regulator in the plasma membrane. Determination of functional half-lives on transfected cells. J Biol Chem 268, 21592–21598 (1993). 7691813

[16] Sharma M, Benharouga M, Hu W, Lukacs GL, Conformational and temperature-sensitive stability defects of the delta F508 cystic fibrosis transmembrane conductance regulator in post-endoplasmic reticulum compartments. J Biol Chem 276, 8942–8950 (2001). 11124952 10.1074/jbc.M009172200

[17] Liu F, Zhang Z, Csanady L, Gadsby DC, Chen J, Molecular Structure of the Human CFTR Ion Channel. Cell 169, 85–95 e88 (2017). 28340353 10.1016/j.cell.2017.02.024

[18] Vergani P, Lockless SW, Nairn AC, Gadsby DC, CFTR channel opening by ATP-driven tight dimerization of its nucleotide-binding domains. Nature 433, 876–880 (2005). 15729345 10.1038/nature03313 PMC2756053

[19] Lewis HA , Impact of the deltaF508 mutation in first nucleotide-binding domain of human cystic fibrosis transmembrane conductance regulator on domain folding and structure. J Biol Chem 280, 1346–1353 (2005). 15528182 10.1074/jbc.M410968200

[20] Rabeh WM , Correction of both NBD1 energetics and domain interface is required to restore DeltaF508 CFTR folding and function. Cell 148, 150–163 (2012). 22265408 10.1016/j.cell.2011.11.024 PMC3431169

[21] Du K, Sharma M, Lukacs GL, The DeltaF508 cystic fibrosis mutation impairs domain-domain interactions and arrests post-translational folding of CFTR. Nat Struct Mol Biol 12, 17–25 (2005). 15619635 10.1038/nsmb882

[22] He L , Restoration of domain folding and interdomain assembly by second-site suppressors of the DeltaF508 mutation in CFTR. FASEB J 24, 3103–3112 (2010). 20233947 10.1096/fj.09-141788 PMC2909275

[23] He L , Restoration of NBD1 thermal stability is necessary and sufficient to correct F508 CFTR folding and assembly. J Mol Biol 427, 106–120 (2015). 25083918 10.1016/j.jmb.2014.07.026 PMC4757845

[24] Rowe SM, Verkman AS, Cystic fibrosis transmembrane regulator correctors and potentiators. Cold Spring Harb Perspect Med 3, (2013). 10.1101/cshperspect.a009761 PMC3685879 23818513

[25] Zaher A, ElSaygh J, Elsori D, ElSaygh H, Sanni A, A Review of Trikafta: Triple Cystic Fibrosis Transmembrane Conductance Regulator (CFTR) Modulator Therapy. Cureus 13, e16144 (2021). 34268058 10.7759/cureus.16144 PMC8266292

[26] Okiyoneda T , Mechanism-based corrector combination restores DeltaF508-CFTR folding and function. Nat Chem Biol 9, 444–454 (2013). 23666117 10.1038/nchembio.1253 PMC3840170

[27] Veit G , Allosteric folding correction of F508del and rare CFTR mutants by elexacaftor-tezacaftor-ivacaftor (Trikafta) combination. JCI Insight 5, (2020). 10.1172/jci.insight.139983 PMC7526550 32853178

[28] Liu F , Structural identification of a hotspot on CFTR for potentiation. Science 364, 1184–1188 (2019). 31221859 10.1126/science.aaw7611 PMC7184887

[29] Fiedorczuk K, Chen J, Mechanism of CFTR correction by type I folding correctors. Cell 185, 158–168 e111 (2022). 34995514 10.1016/j.cell.2021.12.009

[30] Keating D , VX-445-Tezacaftor-Ivacaftor in Patients with Cystic Fibrosis and One or Two Phe508del Alleles. N Engl J Med 379, 1612–1620 (2018). 30334692 10.1056/NEJMoa1807120 PMC6289290

[31] Veit G, Vaccarin C, Lukacs GL, Elexacaftor co-potentiates the activity of F508del and gating mutants of CFTR. J Cyst Fibros 20, 895–898 (2021). 33775603 10.1016/j.jcf.2021.03.011 PMC8463622

[32] Shaughnessy CA, Zeitlin PL, Bratcher PE, Elexacaftor is a CFTR potentiator and acts synergistically with ivacaftor during acute and chronic treatment. Sci Rep 11, 19810 (2021). 34615919 10.1038/s41598-021-99184-1 PMC8494914

[33] Laselva O , Rescue of multiple class II CFTR mutations by elexacaftor+tezacaftor+ivacaftor mediated in part by the dual activities of elexacaftor as both corrector and potentiator. Eur Respir J 57, (2021). 10.1183/13993003.02774-2020 PMC8209484 33303536

[34] Zhang Z, Liu F, Chen J, Conformational Changes of CFTR upon Phosphorylation and ATP Binding. Cell 170, 483–491 e488 (2017). 28735752 10.1016/j.cell.2017.06.041

[35] Zhang Z, Liu F, Chen J, Molecular structure of the ATP-bound, phosphorylated human CFTR. Proc Natl Acad Sci U S A 115, 12757–12762 (2018). 30459277 10.1073/pnas.1815287115 PMC6294961

[36] Chang XB , Role of N-linked oligosaccharides in the biosynthetic processing of the cystic fibrosis membrane conductance regulator. J Cell Sci 121, 2814–2823 (2008). 18682497 10.1242/jcs.028951 PMC2677381

[37] Csanady L, Vergani P, Gadsby DC, Structure, Gating, and Regulation of the Cftr Anion Channel. Physiol Rev 99, 707–738 (2019). 30516439 10.1152/physrev.00007.2018

[38] Jih KY, Li M, Hwang TC, Bompadre SG, The most common cystic fibrosis-associated mutation destabilizes the dimeric state of the nucleotide-binding domains of CFTR. J Physiol 589, 2719–2731 (2011). 21486785 10.1113/jphysiol.2010.202861 PMC3112550

[39] Loo TW, Bartlett MC, Clarke DM, Corrector VX-809 stabilizes the first transmembrane domain of CFTR. Biochem Pharmacol 86, 612–619 (2013). 23835419 10.1016/j.bcp.2013.06.028

[40] Kleizen B , Co-Translational Folding of the First Transmembrane Domain of ABC-Transporter CFTR is Supported by Assembly with the First Cytosolic Domain. J Mol Biol 433, 166955 (2021). 33771570 10.1016/j.jmb.2021.166955

[41] Loo TW, Bartlett MC, Clarke DM, The V510D suppressor mutation stabilizes DeltaF508-CFTR at the cell surface. Biochemistry 49, 6352–6357 (2010). 20590134 10.1021/bi100807h PMC2911077

[42] Krasnov KV, Tzetis M, Cheng J, Guggino WB, Cutting GR, Localization studies of rare missense mutations in cystic fibrosis transmembrane conductance regulator (CFTR) facilitate interpretation of genotype-phenotype relationships. Hum Mutat 29, 1364–1372 (2008). 18951463 10.1002/humu.20866 PMC2785447

[43] Mendoza JL , Requirements for efficient correction of DeltaF508 CFTR revealed by analyses of evolved sequences. Cell 148, 164–174 (2012). 22265409 10.1016/j.cell.2011.11.023 PMC3266553

[44] Thibodeau PH , The cystic fibrosis-causing mutation deltaF508 affects multiple steps in cystic fibrosis transmembrane conductance regulator biogenesis. J Biol Chem 285, 35825–35835 (2010). 20667826 10.1074/jbc.M110.131623 PMC2975206

[45] Capurro V , Partial Rescue of F508del-CFTR Stability and Trafficking Defects by Double Corrector Treatment. Int J Mol Sci 22, (2021). 10.3390/ijms22105262 PMC8156943 34067708

[46] Hoelen H , The primary folding defect and rescue of DeltaF508 CFTR emerge during translation of the mutant domain. PLoS One 5, e15458 (2010). 21152102 10.1371/journal.pone.0015458 PMC2994901

[47] Van Goor F , Correction of the F508del-CFTR protein processing defect in vitro by the investigational drug VX-809. Proc Natl Acad Sci U S A 108, 18843–18848 (2011). 21976485 10.1073/pnas.1105787108 PMC3219147

[48] Lukacs GL, Verkman AS, CFTR: folding, misfolding and correcting the DeltaF508 conformational defect. Trends Mol Med 18, 81–91 (2012). 22138491 10.1016/j.molmed.2011.10.003 PMC3643519

[49] Moniz S , HGF stimulation of Rac1 signaling enhances pharmacological correction of the most prevalent cystic fibrosis mutant F508del-CFTR. ACS Chem Biol 8, 432–442 (2013). 23148778 10.1021/cb300484r

[50] He L , Correctors of DeltaF508 CFTR restore global conformational maturation without thermally stabilizing the mutant protein. FASEB J 27, 536–545 (2013). 23104983 10.1096/fj.12-216119 PMC3545534

[51] Ren HY , VX-809 corrects folding defects in cystic fibrosis transmembrane conductance regulator protein through action on membrane-spanning domain 1. Mol Biol Cell 24, 3016–3024 (2013). 23924900 10.1091/mbc.E13-05-0240 PMC3784376

[52] Naren AP, Quick MW, Collawn JF, Nelson DJ, Kirk KL, Syntaxin 1A inhibits CFTR chloride channels by means of domain-specific protein-protein interactions. Proc. Natl. Acad. Sci. U. S. A 95, 10972–10977 (1998). 9724814 10.1073/pnas.95.18.10972 PMC28005

[53] Naren AP , CFTR chloride channel regulation by an interdomain interaction. Science 286, 544–548 (1999). 10521352 10.1126/science.286.5439.544

[54] Prince LS , Efficient endocytosis of the cystic fibrosis transmembrane conductance regulator requires a tyrosine-based signal. J. Biol. Chem 274, 3602–3609 (1999). 9920908 10.1074/jbc.274.6.3602

[55] Peters KW, Qi J, Johnson JP, Watkins SC, Frizzell RA, Role of snare proteins in CFTR and ENaC trafficking. Pflugers Arch. 443 Suppl 1, S65–69 (2001). 11845306 10.1007/s004240100647

[56] Bilan F , Syntaxin 8 impairs trafficking of cystic fibrosis transmembrane conductance regulator (CFTR) and inhibits its channel activity. J. Cell Sci 117, 1923–1935 (2004). 15039462 10.1242/jcs.01070

[57] Jurkuvenaite A , Mutations in the amino terminus of the cystic fibrosis transmembrane conductance regulator enhance endocytosis. J. Biol. Chem 281, 3329–3334 (2006). 16339147 10.1074/jbc.M508131200

[58] Gene GG , N-terminal CFTR missense variants severely affect the behavior of the CFTR chloride channel. Hum. Mutat 29, 738–749 (2008). 18306312 10.1002/humu.20721

[59] Fu J, Ji HL, Naren AP, Kirk KL, A cluster of negative charges at the amino terminal tail of CFTR regulates ATP-dependent channel gating. J. Physiol 536, 459–470 (2001). 11600681 10.1111/j.1469-7793.2001.0459c.xd PMC2278861

[60] Jisu Im TH, Yeoh Hui Ying, Sahasrabudhe Priyanka, Mijnders Marjolein, van Willigen Marcel, van der Sluijs Peter, Braakman Ineke, ABC-transporter CFTR folds with high fidelity through a modular, stepwise pathway. bioRxiv, (2022). 10.1007/s00018-022-04671-x PMC9825563 36609925

[61] Edelheit O, Hanukoglu A, Hanukoglu I, Simple and efficient site-directed mutagenesis using two single-primer reactions in parallel to generate mutants for protein structure-function studies. BMC Biotechnol 9, 61 (2009). 19566935 10.1186/1472-6750-9-61 PMC2711942

[62] Zheng SQ , MotionCor2: anisotropic correction of beam-induced motion for improved cryo-electron microscopy. Nat Methods 14, 331–332 (2017). 28250466 10.1038/nmeth.4193 PMC5494038

[63] Rohou A, Grigorieff N, CTFFIND4: Fast and accurate defocus estimation from electron micrographs. J Struct Biol 192, 216–221 (2015). 26278980 10.1016/j.jsb.2015.08.008 PMC6760662

[64] Zhang K, Gctf: Real-time CTF determination and correction. J Struct Biol 193, 1–12 (2016). 26592709 10.1016/j.jsb.2015.11.003 PMC4711343

[65] Scheres SH, RELION: implementation of a Bayesian approach to cryo-EM structure determination. J Struct Biol 180, 519–530 (2012). 23000701 10.1016/j.jsb.2012.09.006 PMC3690530

[66] Pettersen EF , UCSF Chimera--a visualization system for exploratory research and analysis. J Comput Chem 25, 1605–1612 (2004). 15264254 10.1002/jcc.20084

[67] Emsley P, Cowtan K, Coot: model-building tools for molecular graphics. Acta Crystallogr D Biol Crystallogr 60, 2126–2132 (2004). 15572765 10.1107/S0907444904019158

[68] Adams PD , PHENIX: a comprehensive Python-based system for macromolecular structure solution. Acta Crystallogr D Biol Crystallogr 66, 213–221 (2010). 20124702 10.1107/S0907444909052925 PMC2815670

[69] Chen VB , MolProbity: all-atom structure validation for macromolecular crystallography. Acta Crystallogr D Biol Crystallogr 66, 12–21 (2010). 20057044 10.1107/S0907444909042073 PMC2803126

[70] Schindelin J , Fiji: an open-source platform for biological-image analysis. Nat Methods 9, 676–682 (2012). 22743772 10.1038/nmeth.2019 PMC3855844

[71] Yeh HI, Sohma Y, Conrath K, Hwang TC, A common mechanism for CFTR potentiators. J Gen Physiol 149, 1105–1118 (2017). 29079713 10.1085/jgp.201711886 PMC5715911

