# Supplementary material for: Molecular Structures Reveal Synergistic Rescue of Δ508 CFTR by Trikafta Modulators
Source: Science. Author manuscript; Available in PMC 2023 Feb 10. (PMC9912939; doi:10.1126/science.ade2216)
Supplement: 1 [file NIHMS1867864-supplement-1.pdf]

Science
AAAS

Supplementary Materials for

Molecular Structures Reveal Synergistic Rescue of Δ508 CFTR by Trikafta Modulators

Karol Fiedorczuk[1] and Jue Chen[1,2,*]

[1] Laboratory of Membrane Biology and Biophysics, The Rockefeller University, New York, NY 10065, USA.

[2] Howard Hughes Medical Institute, Chevy Chase, MD 20815, USA.

[*] Corresponding author. Email: juechen@rockefeller.edu

This PDF file includes:

Materials and Methods
Figs. S1 to S5
Table S1

Materials and Methods

Cell culture

Sf9 cells were cultured in Sf-900 II SFM medium (GIBCO) supplemented with 5% FBS and 1% Antibiotic-Antimycotic. HEK293S GnTI$^-$ cells were cultured in Freestyle 293 (GIBCO) supplemented with 2% FBS and 1% Antibiotic-Antimycotic. HEK293F cells were cultured in DMEM F-12 (ATCC) supplemented with 10% FBS and 1% Antibiotic-Antimycotic. CHO K-1 cells were cultured in DMEM F-12 supplemented with 10% FBS and 1X GlutaMAX (GIBCO).

Mutagenesis

All mutations were introduced using the SPRINP mutagenesis methodology (*61*). Mutagenesis primers were designed to be complementary, be of 15-45 nucleotide length, and contain the mutated bases close to the center. The parental plasmid containing CFTR cDNA was amplified in two separate reactions containing either forward or reverse primers. Next, two single-primer PCR products were combined, denatured (95° C, 5 minutes), and cooled gradually over 5 minutes to 37° C. The sample was digested for 12 hours in 37° C. 3 mL of the digest was added to 50 mL of competent XL1-Blue cells for transformation. The cell suspension was spread on LB/ampicillin plates. After incubating the plates overnight at 37° C, random colonies were picked and expanded. Purified plasmid DNA (QIAGEN Plasmid Kit) was sent for sequencing (Genewiz).

Protein expression

CFTR constructs were expressed and purified as described (*8, 29*). DNA encoding human Δ508/E1371Q CFTR labelled at the C-terminus with an eGFP tag was subcloned into BacMam expression system. Bacmids carrying CFTR constructs were generated in E. Coli DH10Bac cells (Invitrogen). Recombinant baculoviruses were produced and amplified in Sf9 cells. Proteins were expressed in HEK293S GnTI$^-$ cells infected with 10% P4 baculovirus at a density of $2.7x10^6$ cells/ml. Cells were induced with 10 mM sodium butyrate 12 hours after infection and cultured at 30° C for another 48 hours before harvesting. Cell pellets were harvested, frozen in liquid nitrogen, and stored at -80° C.

Protein purification

CFTR constructs were expressed and purified as described previously (*8, 29*), with small modifications. Protein for cryo-EM studies were purified using the following method: Cell pellets were solubilized in buffer containing 1.2% 2,2- didecylpropane-1,3-bis-β-D-maltopyranoside (LMNG) and 0.24% Cholesteryl hemisuccinate (CHS) in lysis buffer (20 mM HEPES-HCl pH 7.4, 1 mM $MgCl_2$, 200 mM NaCl, 20% Glycerol, 1 mM ATP and 1 mM DTT). Next, soluble fraction was separated using centrifugation. CFTR was separated from the supernatant via its C-terminal green fluorescence protein (GFP) tag using GFP nanobody coupled Sepharose Beads (GE Healthcare). Beads were

washed with lysis buffer without glycerol and CFTR was eluted by removing the GFP tag with the PreScission Protease. Next, the Δ508/E1371Q CFTR for the NBD-dimerized conformation sample was treated with protein kinase A (PKA) in the presence of 1 mM ATP/$MgCl_2$. At the final stage, protein was purified with size exclusion chromatography in 0.03% digitonin. The CFTR correctors, if added, were present throughout the entire preparation at the concentration of 1 μM (Figure S1). To purify Δ508/E1371Q CFTR in the NBD-separated conformation, cell pellets were solubilized as described above. Next, nucleotide concentration was decreased to 0.3 mM during the GFP nanobody column wash and further to 0.1 mM during PreScission Protease incubation. Afterwards the protein was dephosphorylated with λ-phosphatase and purified with size exclusion chromatography in 0.03% digitonin buffer containing 2% glycerol but no ATP/$MgCl_2$. Samples for the SPA assays were purified using the same protocol with minor modifications. Wild type (WT) human CFTR was purified in the absence of ATP/$MgCl_2$ and was not treated with protein kinase A (PKA) nor λ-phosphatase (Figure S1).

Cryo-EM grids preparation

Immediately after size exclusion chromatography, CFTR modulators were added at concentrations 10-times that of the protein. For example, if the protein was at 2.5 μM, drugs were added to 25 μM final concentration each. Next, the sample was concentrated to 5 mg/mL (32 μM). The NBD-dimerized CFTR was prepared by incubating with 10 mM ATP, 8 mM $MgCl_2$ on ice for 30 minutes. The NBD-separated sample was prepared by adding EDTA to a final concentration of 2 mM. Right before grids freezing, about 3 mM fluorinated Fos-choline-8 was added to the sample. The sample was vitrified on Quantifoil R 0.6/1 300 mesh Cu grids using Vitrobot Mark IV (FEI).

Cryo-EM data acquisition and processing

Cryo-EM images were collected on a 300 kV Titian Krios (FEI) with a K3 Summit detector (Gatan) using SerialEM. The images were corrected for gain reference and binned by 2. Drift correction was performed using MotionCorr (*62*). Contrast transfer function (CTF) estimation was performed using CTFFIND4 (*63*) and GCTF (*64*). The results from CTFFIND4 were used to remove images with a resolution lower than 5 Å. CTF values generated from GCTF were used for subsequent data processing performed using RELION 3.1(*65*) (Figure S2). Processing parameters for each dataset were optimized individually to obtain the best results. In general, after particles extraction a 2D classification was performed. Already at this stage it was possible to assess conformation of NBDs. Next, particles from the best-looking classes were 3D classified. After visual inspection, the most complete classes were picked and used for 3D refinement. If the resulting map showed a well-resolved NBD1, particles were further CTF refined, polished, and 3D refined. Next, they were 3D classified without angles optimization and the best class was 3D refined again to produce the final map. The resolution was estimated using the RELION “golden standard” FSC=0.143 cutoff from the last refinement job for each dataset, with the exception of the Δ508/E1371Q CFTR

with ATP/$MgCl_2$, which was reported by RELION to be at 6 Å resolution. Upon visual inspection of the map and examination of FSC curves profiles, we decided that the resolution estimation was inflated and reported resolution for this map to be ~9 Å.

Model building and refinement

The initial protein models were built by fitting the published CFTR structures (PDB:7SVR and 7SVD) into the cryo-EM maps using UCSF Chimera (*66*). Models were then adjusted based on the cryo-EM densities using Coot (*67*). The model for the Δ508/E1371Q CFTR in the NBD-separated conformation was trimmed to beta-carbons due to low resolution. Elexacaftor, lumacaftor, tezacaftor, and ivacaftor were built into the drug density using restrains generated by Grade Web Server (http://grade.globalphasing.org/). Finally, models were refined in PHENIX (*68*) and validated in MolProbity (*69*).

Maturation assay

The experiments were performed in the same way as described previously (*29*). HEK293F cells grown in a 6-well plate were transiently transfected with CFTR constructs labeled with C-terminal eGFP tag using Lipofectamine 3000 (ThermoFisher) in Opti-MEM (GIBCO) medium. Cells were incubated with DNA/transfection mixture for 12 hours at 37° C, then in DMEM F-12 supplemented with 10 mM sodium butyrate and the corrector of choice at 30° C for another 24 hours. CFTR modulators were added at the following concentrations: lumacaftor 1 μM, tezacaftor 10 μM, elexacaftor 0.5 μM. All the conditions contain 0.1% DMSO. Cells were harvested by re-suspending in 1 mL ice-cold PBS and spun down in 1.5 mL tubes for 5 minutes at 5,000 rpm, 4° C.

Cell pellets were re-suspended in buffer containing 1.2% 2,2- didecylpropane-1,3-bis-β-D-maltopyranoside (LMNG) and 0.24% Cholesteryl hemisuccinate (CHS) and rotated for 60 minutes at 4° C. Cell lysates were spun down for 60 minutes at 45,000 rpm, supernatants were analyzed on a 4-20% gradient tris-glycine SDS-PAGE gel (ThermoFisher). Gels were imaged to visualize the GFP signal, which was quantified using Fiji (*70*). The background signal was subtracted from the CFTR bands. The proportion of mature CFTR to total CFTR ($k_{m/t}$) was calculated using equation 1 and then normalized to that of the DMSO treated sample. Signal from the WT CFTR sample was normalized to that of DMSO treated Δ508 sample.

$$k_{m/t} = \frac{CFTR_{mature}}{(CFTR_{mature} + CFTR_{immature})} \quad (1)$$

Scintillation proximity assay

The binding and competition assays were performed as described (*28, 29*). CFTR constructs used in this assay contain a C-terminal Strep-tag, followed by a PreScission Protease cleavage site, and a GFP tag. The GFP tag was removed during purification whereas the Strep-tag was retained to attach CFTR to the SPA beads. To measure

elexacaftor binding, 5 nM CFTR was incubated with 0.5 mg/ml YSi streptavidin SPA beads (PerkinElmer) in the presence of varying concentrations of elexacaftor at a 1:1 molar ratio of cold and [^{3}H] elexacaftor (5.5 Ci/mmol, synthesized by Moravek) in buffer containing 20 mM Tris-HCl pH 7.5, 200 mM NaCl, 0.06% digitonin, 2 mM DTT and 0.1% Tween 20 at 4° C for 1 hour. The reactions were carried out in 96-well non-binding surface microplates (ThermoFisher Scientific Nunc ref. 265302) and data were recorded using a Microbeta Trilux plate reader (PerkinElmer). Specific binding was obtained by subtracting background radioligand binding in the absence of protein. The K_d values were calculated by fitting the data with a single-site saturation binding model accounting for ligand depletion using GraphPad Prism 9 (GraphPad Software, San Diego, California, USA, www.graphpad.com). The readings were normalized by dividing the specific binding with the total binding (Bmax) and represented in percentages.

Confocal Laser Scanning Microscopy (CLSM) imaging

Confocal analysis was performed as described (*29*). CHO cells were seeded in Ibidi µ-Slide 4 Well Ph+ chambered coverslips and cultured in CHO media. After 24 hours at 37° C, cell media was exchanged for OpitMEM and cells were transiently transfected with mCherry tagged Tapasin and eGFP tagged CFTR isoforms using Lipofectamine 3000 according to the manufacturer’s instructions. About 12 hours after transfection, the media was exchanged back to CHO cell media supplemented with 10 mM sodium butyrate and selected drug (lumacaftor at 1 μM, tezacaftor at 10 μM, or elexacaftor at 0.5 μM). DMSO (0.1%) was included in all samples. Cells were then incubated at 30° C for 24 hours and fixed with 2% paraformaldehyde (15 minutes at room temperature), stained with Alexa Fluor 647- conjugated wheat germ agglutinin (15 minutes at room temperature), and mounted with glycerol/n-propyl gallate. Imaging was performed using the Abberior Facility Line STED/confocal system with a 100X/1.40 NA oil objective (Olympus UPLSAPO100XO objective, oil r.i. 1.518 at 23° C) on an Olympus IX83 stand, pulsed 405 / 485 / 561 / 640 nm excitation laser lines, and spectral detection setup with single-photon-counting avalanche photodiode (APD) detectors. Pixel sizes and z-steps were computed to satisfy Nyquist criteria. Images were acquired as .obf file format using Abberior Imspector software, which automatically adjusted the detection bands (eGFP:498-551nm, mCherry:571-630nm, Alexa Fluor 647:650-760nm) and the confocal pinhole (1 A.U) based on selected wavelengths. Pixel saturation and bleaching were avoided by optimizing laser power, accumulation, and pixel dwell times, while trying to collect an optimal number of photons. Acquisition parameters were kept constant between images. Post-acquisition, images were further processed and analyzed in Fiji.

Inside-out patch clamp recording

The recordings were performed in a similar way as described (*28*). CHO cells were plated at 0.4x10^6 cells per 35-mm dish (Falcon) and grown for 24 hours before transiently transfecting with eGFP-tagged CFTR constructs using Lipofectamine 3000 (ThermoFisher) in Opti-MEM (GIBCO) medium. Cells were incubated with DNA/transfection mixture for 12 hours at 37° C, then in DMEM F-12 supplemented with 3 mM sodium butyrate at 30° C for another 24 hours. Currents were recorded using the

inside-out patch configuration with local perfusion at the patch and using buffer compositions and recording parameters as described (*71*). The culture media were first changed to a bath solution consisting of 145 mM NaCl, 2 mM MgCl2, 5 mM KCl, 1 mM CaCl2, 5 mM glucose, 5 mM HEPES, and 20 mM sucrose, pH 7.4 with NaOH. The pipette solution contains 140 mM NMDG, 5 mM CaCl2, 2 mM MgCl2, and 10 mM HEPES (pH 7.4 with HCl). The perfusion solution contains 150 mM NMDG, 2 mM MgCl2, 1 mM CaCl2, 10 mM EGTA, and 8 mM Tris (pH 7.4 with HCl). Borosilicate micropipettes (OD 1.5 mm, ID 0.86 mm, Sutter) were pulled to 1.5 - 2.5 MΩ resistance and fire-polished. After a gigaseal was formed, inside-out patches were excised and exposed to 25 units/ml PKA (Sigma-Aldrich) and 3 mM ATP to phosphorylate CFTR. In subsequent perfusions, 3 mM ATP was used to open the channel. Currents were recorded at -30 mV and 25° C using an Axopatch 200B amplifier, a Digidata 1550 digitizer, and pCLAMP software (Molecular Devices). The recordings were low-pass filtered at 1 kHz and digitized at 20 kHz. Data were analyzed with Clampfit and GraphPad Prism. For the recordings, we used 10 µM GLPG1837 and 1 µM elexacaftor. For the statistical analysis, currents were normalized to the current at 3 mM ATP without modulators. Fold of current increase upon stimulation with CFTR modulators was calculated and plotted.

Statistical analysis

The P values for maturation assays and electrophysiology experiments were calculated using a paired t-test.

Key reagents sources

CFTR modulators used in the study were purchased or synthesized from the following suppliers:
(R/S) Elexacaftor (VX-445) for the cryo-EM studies from Kalexsyn (synthesized to order)
(R/S) Elexacaftor (VX-445) for the SPA assay from Selleckchem (S8851) and radiolabelled with [H^3] by Moravek.
(R) Elexacaftor (VX-445) from MedChemExpress (HY-111772A)
(S) Elexacaftor (VX-445) from MedChemExpress (HY-111772)
Tezacaftor from MedChemExpress (HY-15448)
Lumacaftor from MedChemExpress (HY-13262)
GLPG1837 from MedChemExpress (HY-111099)
ATP from Sigma Aldrich (A2383)

Data Presentation

Structural figures were generated using UCSF Chimera, ChimeraX, PyMOL, and Fiji. Plots were generated using GraphPad Prism9.

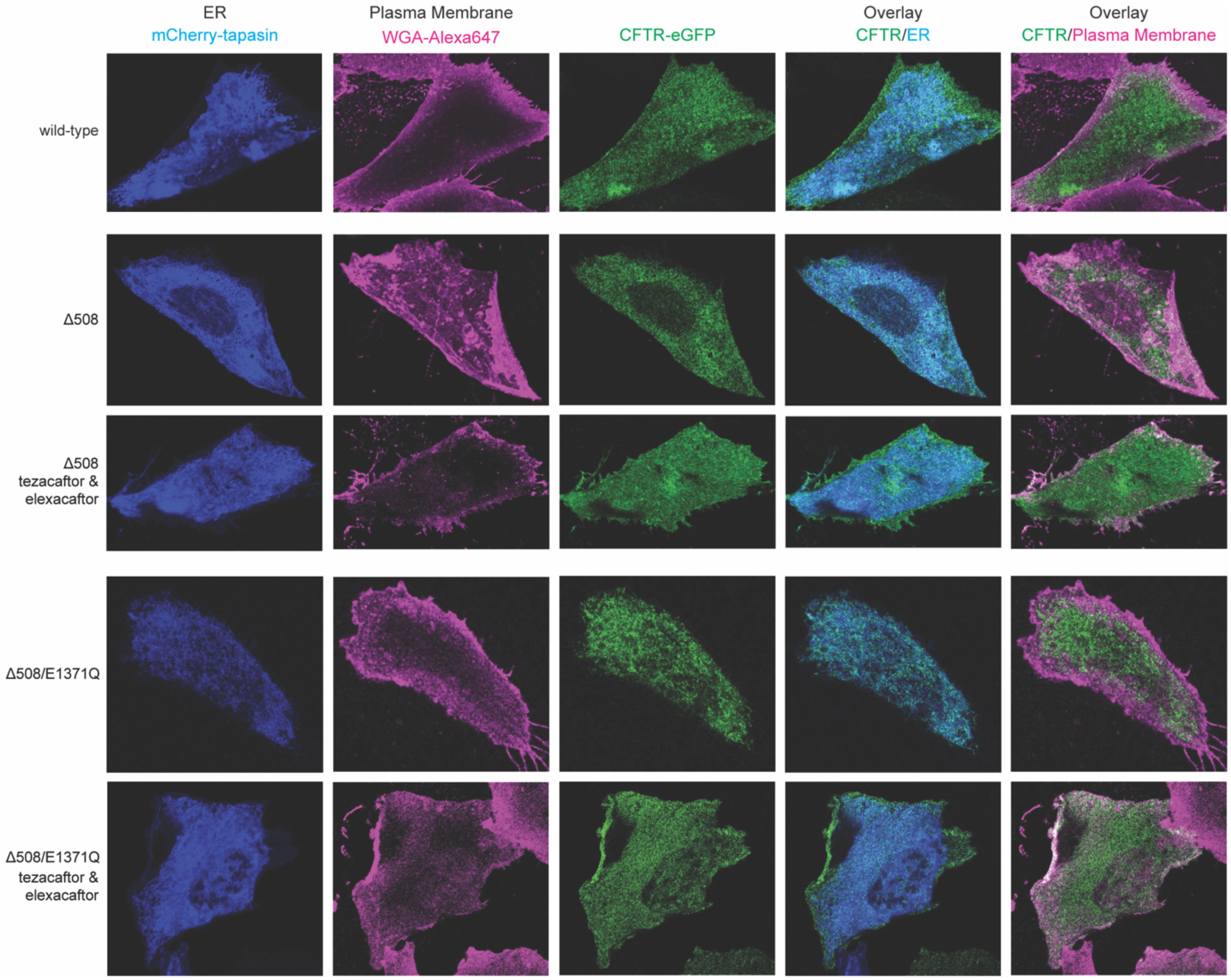

Figure S1. Confocal Laser Scanning Microscopy analysis of CFTR variants

Cells expressing CFTR variants were treated with DMSO or tezacaftor/elexacaftor cocktail. ER (red) is visualized via the mCherry fused to Tapasin. Plasma membrane (magenta) is visualized by exciting Alexa Fluor 647- conjugated wheat germ agglutinin staining. CFTR (green) is visualized via eGFP fused to CFTR. The first three columns show the signal from every excitation/emission channel separately. The last two columns show the overlayed signals.

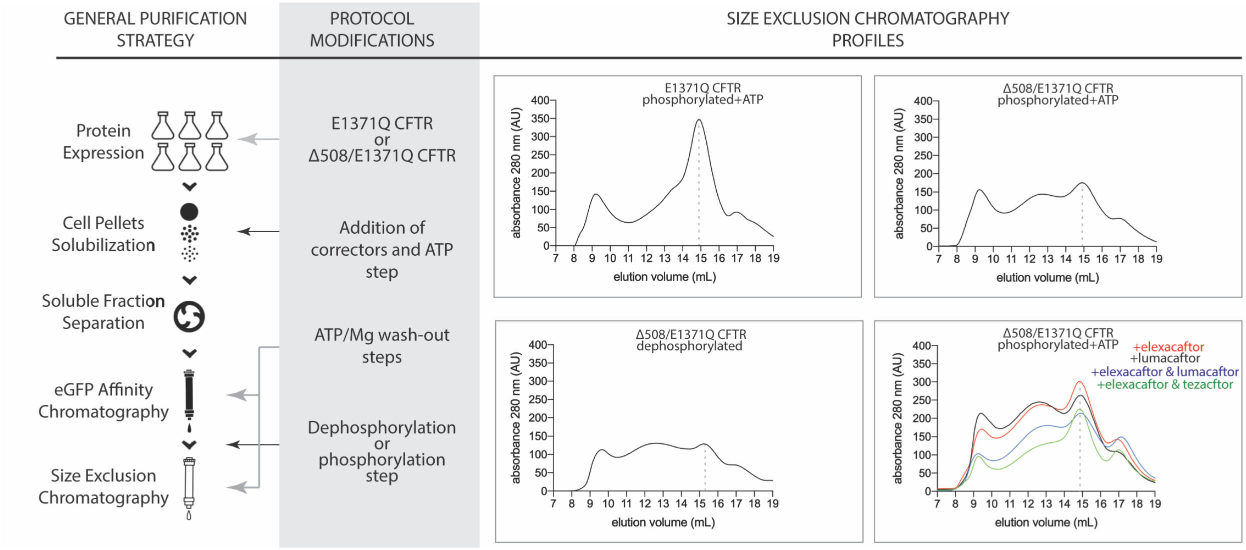

Figure S2. Purification of different CFTR constructs

Left: Flowchart showing the general strategy used to purify the CFTR variants used in this study. Correctors and ATP/Mg^{2+} were added during membrane solubilization. Protein was dephosphorylated or phosphorylated prior to size exclusion chromatography. Details are described in methods.

Right: Size exclusion chromatography profiles of the protein samples used for structural analysis. The E1371Q CFTR profile is included as a reference and was not analyzed structurally in this study. The dashed line marks the position of the elution fraction used for grids preparation.

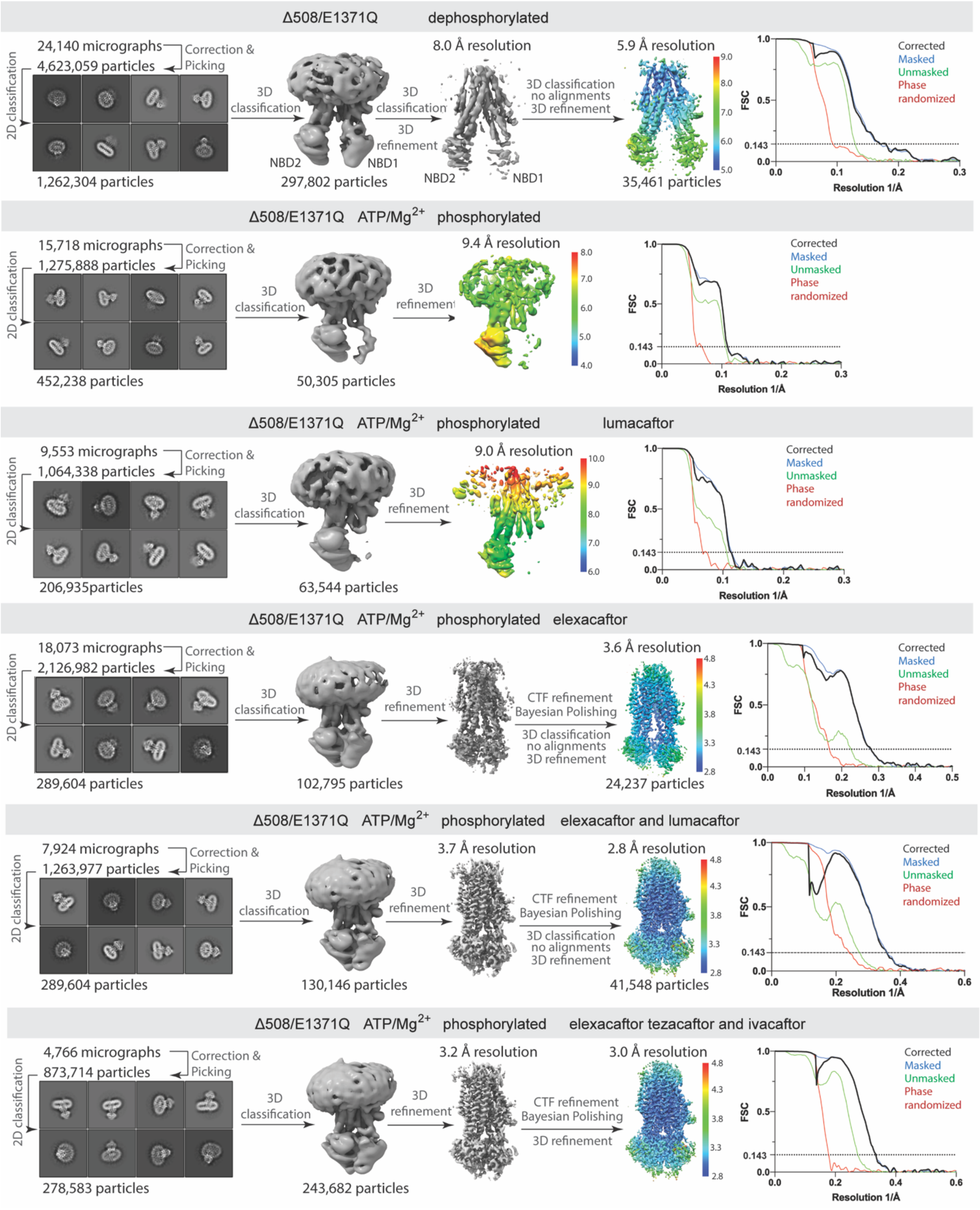

Figure S3. Summary of cryo-EM data collection and processing of the six structures presented in this study

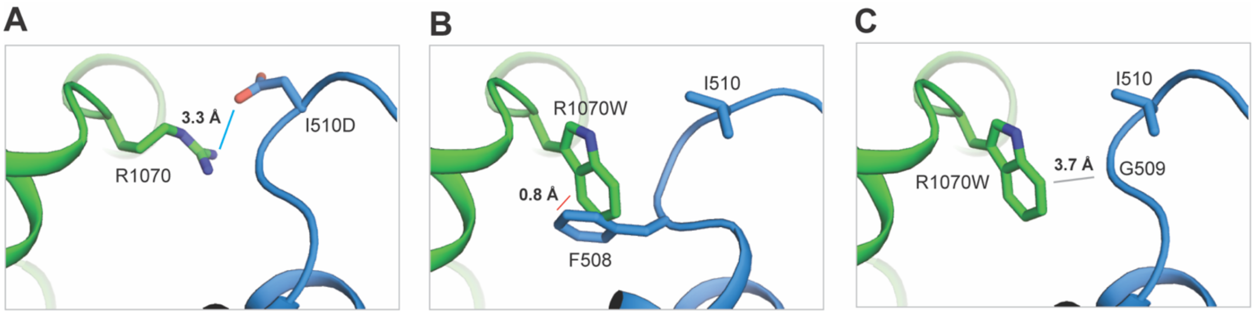

Figure S4. Modelling of the F508 site based on published mutagenesis data

(A) Structure of Δ508/I510D. Introducing I510D in the background of the Δ508 CFTR likely stabilizes the NBD1/TMD1 interface by forming an electrostatic interaction with the guanidino group of R1070 as suggested (39).
(B) Introducing R1070W in the background of full-length CFTR likely results in steric clashes with F508.
(C) Structure of R1070W in the background of the Δ508 CFTR, the introduced large indole ring likely stabilizes the NBD1/TMD1 interface by filling the void created by F508 deletion.

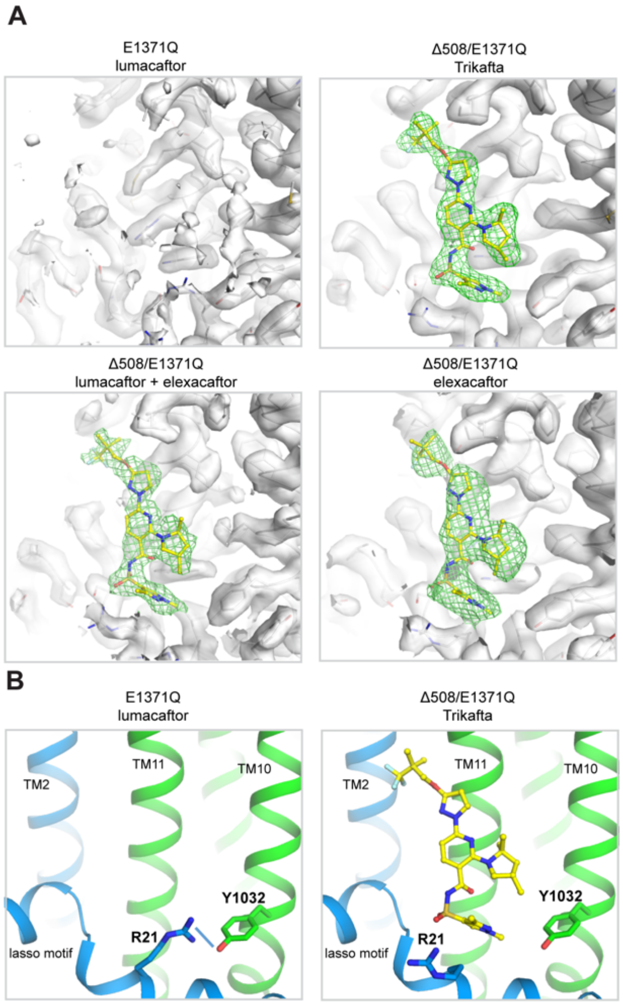

Figure S5. The elexacaftor binding site.

(A) Cryo-EM maps obtained in the absence and presence of elexacaftor. Protein is represented as grey ribbons and sticks, elexacaftor as in sticks model. The protein density is represented as grey semi-transparent surface and the density of the drug is represented as green mesh.
(B) Local conformational changes of R21 upon elexacaftor binding. In the absence of elexacaftor (Left panel, PDB: 7SVD), R21 and Y1032 form an H-bond. In the presence of elexacaftor, R21 repositions to interact with elexacaftor (Right panel).

Table S1. Cryo-EM data collection, refinement, and validation statistics

	Δ508/E1371Q Dephosphorylated no ATP/Mg^{2+} no modulators PDB 8EJ1 EMD-28172	Δ508/E1371Q Phosphorylated ATP/Mg^{2+} no modulators PDB N/A EMDB N/A	Δ508/E1371Q Phosphorylated ATP/Mg^{2+} lumacaftor PDB N/A EMDB N/A	Δ508/E1371Q Phosphorylated ATP/Mg^{2+} elexacaftor PDB 8EIG EMD-28155	Δ508/E1371Q Phosphorylated ATP/Mg^{2+} elexacaftor lumacaftor PDB 8EIO EMD-28160	Δ508/E1371Q Phosphorylated ATP/Mg^{2+} elexacaftor tezacaftor ivacaftor PDB 8EIQ EMD-28161
**Data collection and processing**						
Microscope	Titan Krios	Titan Krios	Titan Krios	Titan Krios	Titan Krios	Titan Krios
Camera	Gatan K3	Gatan K3	Gatan K3	Gatan K3	Gatan K3	Gatan K3
Automation software	SerialEM	SerialEM	SerialEM	SerialEM	SerialEM	SerialEM
Magnification	105,000	135,00	105,000	105,000	105,000	105,000
Voltage (kV)	300	300	300	300	300	300
Total electron exposure ($e^-/Å^2$)	65.6	73.5	59.1	65.6	65.6	65.6
Exposure rate (e^-/pix/s)	10	13	15	25	20	20
Total frames	50	50	45	40	50	50
Defocus range (μm)	0.8 – 1.8	0.8 - 1.8	0.8 – 1.8	0.8 - 1.8	0.8 – 1.8	0.8 - 1.8
Pixel size (Å)	0.676	0.515	0.676	0.676	0.676	0.676
Symmetry imposed	C1	C1	C1	C1	C1	C1
Micrographs collected	24,140	15,718	9,553	18,073	7,924	4,775
Initial particle images (no.)	4,623,059	1,275,888	1,064,338	2,126,982	873,714	1,263,977
Final particle images (no.)	35,461	50,305	63,544	24,237	41,548	243,682
Symmetry	C1	C1	C1	C1	C1	C1
Map resolution masked (Å)	5.9	9.4	9.0	3.6	2.8	3.0
FSC threshold	0.143	0.143	0.143	0.143	0.143	0.143
Map resolution range (Å)	5.0 – 8.0	8.0 - 10.0	8.0 - 10.0	3.5 – 4.5	2.8 – 3.5	2.8 – 3.8
Map sharpening B factor	-241.0	-695.1	-528.7	-63.4	-30.0	-67.5
Map sharpening method	global	global	global	global	global	global
**Refinement**						
Initial model used (PDB code)	7SVR	N/A	N/A	7SVD	7SVD	7SVD
Refinement Package	Phenix	N/A	N/A	Phenix	Phenix	Phenix
Model resolution (Å)	7.6	N/A	N/A	3.7	3.1	3.2
FSC threshold	0.5			0.5	0.5	0.5
Model resolution range (Å)	5.0 – 8.0	N/A	N/A	3.5 – 4.5	2.8 – 3.5	2.8 – 3.8
Model composition		N/A	N/A			
Non-hydrogen atoms	5680			9282	9557	9597
Protein residues	1124			1152	1181	1182
Ligands	0			6	8	9
CC masked	0.73	N/A	N/A	0.79	0.86	0.87
B factors ($Å^2$) mean		N/A	N/A			
Protein	90.6			27.9	59.9	71.3
Ligands	N/A			45.3	56.2	58.8
R.m.s. deviations		N/A	N/A			
Bond lengths (Å)	0.001			0.003	0.002	0.003
Bond angles (°)	0.27			0.9	0.9	0.9
Validation		N/A	N/A			
MolProbity score	1.08			1.3	0.82	1.0
Clashscore	0.6			2.0	1	1.1
Poor rotamers (%)	0.0			1.9	1.1	1.8
CaBLAM outliers	1.9			1.9	0.69	1.2
Ramachandran plot		N/A	N/A			
Favored (%)	97.8			97.4	99.0	99.0
Allowed (%)	1.9			2.5	0.9	1.3
Disallowed (%)	0.3			0.1	0.1	0.0
